# Gammaretroviral *pol* sequences act *in cis* to direct polysome loading and NXF1/NXT-dependent protein production by *gag*-encoded RNA

**DOI:** 10.1186/s12977-014-0073-0

**Published:** 2014-09-12

**Authors:** Hanni Bartels, Jeremy Luban

**Affiliations:** Department of Microbiology and Molecular Medicine, University of Geneva, Geneva, 1205 Switzerland; Program in Molecular Medicine, University of Massachusetts Medical School, Worcester, MA 01605 USA

## Abstract

**Background:**

All retroviruses synthesize essential proteins via alternatively spliced mRNAs. Retrovirus genera, though, exploit different mechanisms to coordinate the synthesis of proteins from alternatively spliced mRNAs. The best studied of these retroviral, post-transcriptional effectors are the *trans*-acting Rev protein of lentiviruses and the *cis*-acting constitutive transport element (CTE) of the betaretrovirus Mason-Pfizer monkey virus (MPMV). How members of the gammaretrovirus genus translate protein from unspliced RNA has not been elucidated.

**Results:**

The mechanism by which two gammaretroviruses, XMRV and MLV, synthesize the Gag polyprotein (Pr65^Gag^) from full-length, unspliced mRNA was investigated here. The yield of Pr65^Gag^ from a *gag*–only expression plasmid was found to be at least 30-fold less than that from an otherwise isogenic *gag-pol* expression plasmid. A frameshift mutation disrupting the *pol* open reading frame within the *gag-pol* expression plasmid did not decrease Pr65^Gag^ production and 398 silent nucleotide changes engineered into *gag* rendered Pr65^Gag^ synthesis *pol*-independent. These results are consistent with *pol-*encoded RNA acting in *cis* to promote Pr65^Gag^ translation. Two independently-acting *pol* fragments were identified by screening 17 *pol* deletion mutations. To determine the mechanism by which *pol* promoted Pr65^Gag^ synthesis, *gag* RNA in total and cytoplasmic fractions was quantitated by northern blot and by RT-PCR. The *pol* sequences caused, maximally, three-fold increase in total or cytoplasmic *gag* mRNA. Instead, *pol* sequences increased *gag* mRNA association with polyribosomes ~100-fold, a magnitude sufficient to explain the increase in Pr65^Gag^ translation efficiency. The MPMV CTE, an NXF1-binding element, substituted for *pol* in promoting Pr65^Gag^ synthesis. A *pol* RNA stem-loop resembling the CTE promoted Pr65^Gag^ synthesis. Over-expression of NXF1 and NXT, host factors that bind to the MPMV CTE, synergized with *pol* to promote gammaretroviral *gag* RNA loading onto polysomes and to increase Pr65^Gag^ synthesis. Conversely, Gag polyprotein synthesis was decreased by NXF1 knockdown. Finally, overexpression of SRp20, a shuttling protein that binds to NXF1 and promotes NXF1 binding to RNA, also increased *gag* RNA loading onto polysomes and increased Pr65^Gag^ synthesis.

**Conclusion:**

These experiments demonstrate that gammaretroviral *pol* sequences act *in cis* to recruit NXF1 and SRp20 to promote polysome loading of *gag* RNA and, thereby license the synthesis of Pr65^Gag^ from unspliced mRNA.

## Background

Retroviruses compress large quantities of genetic information into their relatively small genomes. HIV-1, for example, has a single promoter that drives a primary transcript, from which 9 genes direct the synthesis of at least 15 proteins [1,2]. This is accomplished by exploiting several mechanisms, including the synthesis of essential viral proteins from unspliced or partially spliced mRNAs [1,3,4]. In all retroviruses, the primary, unspliced transcript serves as the viral genomic RNA that is packaged into assembling virions. Unspliced RNA of identical primary sequence also directs translation of the main virion structural elements, the *gag*–encoded proteins [5]. Assessment of HIV-1 transcripts by conventional methods has revealed nearly 50 variants [6], while newer deep sequencing technology has detected more than 100 [7]. Replication of HIV-1 and other retroviruses must therefore necessitate an exquisite balance of these differentially spliced mRNAs [8].

Unspliced and or incompletely spliced RNAs are generally retained in the nucleus until splicing is completed, though the spliceosome is increasingly appreciated to function as a highly dynamic machine [9]. Therefore, in order to generate viral proteins essential for virus replication, retroviruses must encode *cis*-acting RNA sequences that recruit *trans*-acting cellular factors, and, in some cases, *trans*-acting viral factors. Different classes of retroviruses have evolved unique mechanisms for exporting unspliced mRNAs out of the nucleus in such a way that they are efficiently translated. The lentivirus HIV-1, for example, encodes a *trans*-acting protein, Rev, that binds to a *cis*-acting RNA sequence in the unspliced mRNA called the Rev-Response Element (RRE); Rev then links the RNA to the CRM1-mediated export pathway [2,10-12]. In analogous fashion, the *cis*-acting constitutive transport element (CTE) in the betaretrovirus Mason-Pfizer monkey virus (MPMV) mRNA recruits the cellular export factor NXF1 [13-16]. Other betaretroviruses, including Jaagsiekte sheep retroviruse (JSRV), mouse mammary tumor virus (MMTV), and human endogenous retrovirus type K (ERV-K), additionally encode a *trans*-acting factor necessary for Gag protein production [17,18]. The alpha-retrovirus Rous sarcoma virus (RSV) possesses two direct repeat (DR) sequences that function as CTE-like elements, though one copy is sufficient to provide translocation into the cytoplasm [19-21]. Avian leukosis virus (ALV) possesses a single DR in its 3’UTR region [20,22].

How unspliced gammaretroviral mRNAs are stabilized, exported from the nucleus, and translated into protein is not known. In the course of developing expression vectors for gammaretroviruses, we observed that *gag* coding sequences in isolation were unable to direct the synthesis of *gag*-encoded protein. This observation prompted investigation of post-transcriptional regulation in gammaretroviruses.

## Results

### Gammaretrovirus *pol* increases the levels of the Gag polyprotein

**Figure 1:**
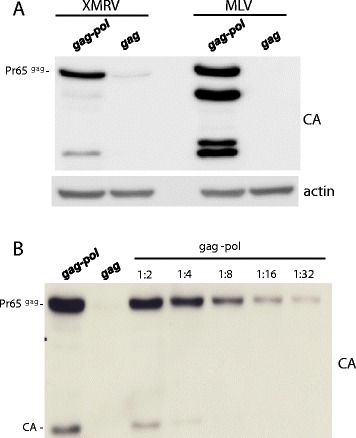
**Gammaretroviral** ***pol*** **sequence is required for efficient Pr65** ^**Gag**^ **protein production. (A)** HEK293T cells were transfected with the indicated XMRV and MLV expression constructs. Cell lysate was harvested 48 hrs later and probed with anti-CA antibody (upper panel) or anti-β-actin antibody (lowel panel). **(B)** The magnitude difference in XMRV Pr65^Gag^ protein level in cells tranfected with the the *gag-pol* or *gag*-only expression plasmids was determined by comparing the *gag*-only signal with serial dilutions of lysate from cells transfected with *gag-pol*.

In the course of generating minimal retroviral vectors using genes from XMRV and MLV, the *gag*-*pol* sequences of each were placed under the control of the cytomegalovirus immediate early promoter (CMV_IE_). HEK293T cells were transfected with the XMRV or MLV *gag-pol* plasmids. The cell lysates were collected 48 hrs later and probed with anti-CA antibody (upper panel) or anti-β-actin antibody as a loading control (lowel panel). The transfection of either XMRV *gag-pol* (Figure 1A, left), or of MLV *gag-pol* (Figure 1A, right), resulted in Gag protein production in the cell lysate that was clearly detectable by this method. Additionally, when co-transfected with plasmids encoding vesicular stomatitis virus glycoprotein (VSV G) and a packageable MLV-GFP reporter genome, either XMRV or MLV *gag-pol* construct produced reverse transcriptase (RT)-positive particles in the supernatant that could be pelleted by ultracentifugation; the XMRV and MLV particles transduced GFP into HEK293T cells at comparable efficiency.

In parallel with these experiments, the *gag* open reading frame from XMRV or MLV was cloned into identical expression plasmids in the absence of any *pol* sequences. When either the XMRV *gag* expression plasmid (Figure 1A, left panel) or the MLV *gag* expression plasmid (Figure 1A, right panel) were transfected into 293 T cells, to our surprise, Gag protein production was difficult to detect by western. Gag polyprotein production, then, was inefficient in the absence of *pol*.

To quantify the difference in the level of the XMRV Gag polyprotein, lysate from cells transfected with the *gag*-only expression plasmid was compared with serial dilutions of lysate from cells transfected with *gag-pol*. 48 hrs after 293 T cell transfection the lysates were probed in an immunoblot with a CA-specific antibody. The Gag polyprotein signal was at least 30-fold greater with *gag-pol* than it was with *gag-*only (Figure 1B).

### The gammaretrovirus *pol* gene acts at the level of RNA to promote synthesis of the Gag polyprotein

**Figure 2:**
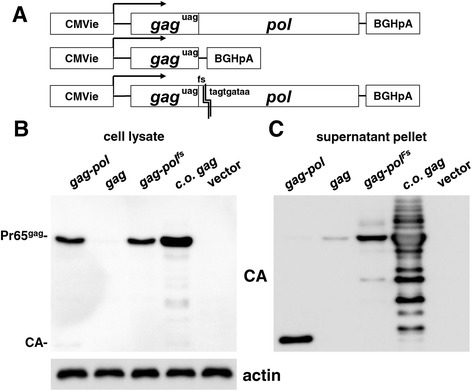
**Protein synthesis by** ***pol*** **is not required to promote Pr65** ^**Gag**^ **protein production.** HEK293T cells were transfected with the indicated constructs (XMRV *gag-pol*, XMRV *gag*, XMRV *gag-pol* with a frameshift mutation just after the XMRV *gag* stop codon, codon optimized XMRV *gag*, or empty vector), and harvested 48 hrs later. **(A)** Schematic representation of XMRV constructs showing interruption of *pol* translation by introduction of a frameshift mutation just after the stop codon of XMRV *gag*. **(B)** HEK293T cell lysate was probed with anti-CA antibody (upper panel) and anti-β-actin antibody (lowel panel). **(C)** Virus-like particles (VLPs) pelleted from the supernatant by ultracentrifugation were collected and analyzed by immunoblotting with anti-CA antibody.

The gammaretrovirus *pol* gene is in the same reading frame as *gag*, immediately 3’ of the *gag* UAG stop codon. Translation of the *pol* open reading frame requires read-through of the *gag* UAG stop codon such that the ribosome incorporates a glutamine to generate a Gag-Pol fusion protein [23] (Figure 2A). To determine if translation of *pol* is required for Gag polyprotein synthesis a *gag-pol* expression plasmid was engineered that bears a frameshift mutation at the beginning of the *pol* open reading frame (Figure 2A). The frameshift mutation renders *pol* out-of-frame with the consequence that stop codons are soon encountered and *pol* translation terminates prematurely.

The *gag-pol* expression plasmid containing the frameshift mutation was transfected into 293 T cells, in parallel with the wild-type *gag-pol* and *gag*-only expression plasmids. 48 hrs later, cell lysate was analyzed by immunoblot using anti-CA antibody (upper panel) or anti-β-actin antibody (lowel panel) (Figure 2B). Gag protein production by the *gag-pol* frameshift plasmid was indistinguishable from that of the wild-type *gag-pol* plasmid. This indicates that the *pol* sequence acts at the level of the RNA, and that it need not be translated into protein, to stimulate Gag polyprotein production.

The fact that *pol* RNA acted in *cis* to promote Gag polyprotein production suggested that the basis for the deficiency in Gag polyprotein production by the *gag*-alone plasmid would be at the level of *gag* RNA. To test if this was the case, a codon-optimized *gag* open reading-frame was generated and cloned into an isogenic expression plasmid and tested for its ability to generate Gag polyprotein in the absence of *pol*. The codon optimized *gag* has 398 silent nucleotide changes with respect to the original *gag* sequence and changes the GC content from 55% to 64%. When transfected in parallel with the other plasmids, the codon-optimized *gag* sequence increased Gag protein production well beyond the levels produced by the *gag-pol* plasmid (Figure 2B and C). This indicates that the relatively low levels of Gag polyprotein result from a deficiency at the level of *gag* RNA and that it does not result, for example, from protein instability.

The relatively low steady-state level of Gag polyprotein in the lysate of cells transfected with the *gag*-only plasmid might also be a consequence of Gag polyprotein budding off from the producer cell at a rate that exceeds the ability of the cell-associated protein to accumulate to detectable levels. To determine if this was the case, the supernatant was collected 48 hrs after transfection of the 293 T cells with the *gag* only and *gag-pol* expression plasmids. The supernatant was accelerated through a 25% sucrose cushion and protein in the pellet was immunoblotted with a CA-specific antibody (Figure 2C). The relative intensity of the Gag signal in the pellet tracked with the intensity of the signal in the cell lysate. This result indicates that the lower Gag polyprotein levels in the lysate of cells transfected with the *gag*-only plasmid did not result from an accelerated rate of virion budding and release.

### Mapping of *pol* sequence required to promote Gag polyprotein production

The previous experiments demonstrated that *pol* sequences act at the level of the RNA to promote Gag polyprotein production. To map the *pol* sequences responsible for this activity an expression plasmid was engineered that contains XMRV *gag* followed by *pol* sequences, in such a way that translation of the *pol* sequences was precluded. Normally gammaretrovirus *pol* is expressed by read-through suppression of the *gag* UAG stop codon [23]. The *gag* UAG stop codon in this construct was replaced with a UGA stop codon and the *pol* sequences were cloned such that they were out-of-frame with respect to *gag*. Either modification alone is sufficient to prevent *pol* translation.

**Figure 3:**
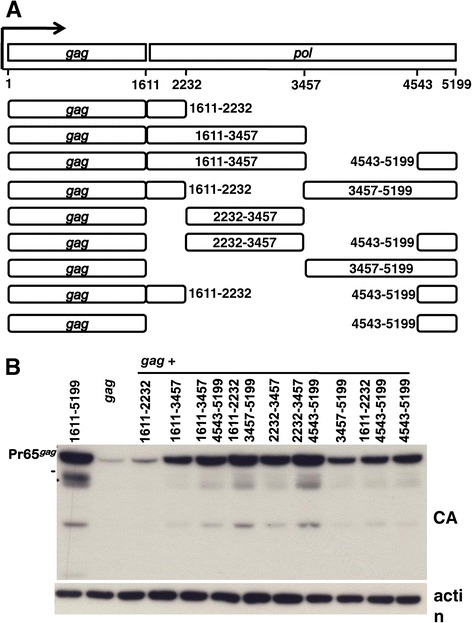
**Either of two** ***pol*** **fragments promote Gag protein production.** HEK293T cells were transfected with the indicated constructs and cell lysate was harvested 48 hrs later. **(A)** Schematic of the XMRV *pol* sequence fragments that were cloned out of frame and downstream of XMRV *gag* in which the natural UAG stop codon was replaced with UGA. **(B)** Cell lysate was probed with anti-CA antibody (upper panel) and anti-β-actin antibody (lowel panel).

Unique restriction sites within *pol* were used to generate *pol* fragments and deletions that were cloned 3’ of the *gag* open reading frame (Figure 3A). The *pol* open reading frame is between nucleotides 1611 and 5199. The *pol* mutants were named with respect to the nucleotide numbers of the *pol* sequences that they retained. Each construct was transfected into 293 T cells in parallel with the *gag-pol* and *gag*-only expression plasmids. Cell lysate from each transfection was analyzed by immunoblotting with anti-CA antibody and anti-β-actin antibody as a loading control.

To varying degrees, each of the nine engineered *pol* fragments that were tested increased Gag protein production above the level observed with *gag* alone (Figure 3B). Of the fragments tested, the 1611–2232 fragment had the smallest effect, only increasing Gag protein levels about 2-fold. Either of two *pol* fragments, 2232–3456 or 4543–5199, caused a large increase in Gag protein production. When combined together, the effect of 2232–3456 and 4543–5199 was additive, increasing Gag protein to the same level as the complete *pol*. These mapping results indicate that there are two regions within *pol* that are each independently capable of promoting Gag protein production, and that the two regions act together for the full effect (Figure 3B).

### Mapping *pol* fragment 2232–3456

**Figure 4:**
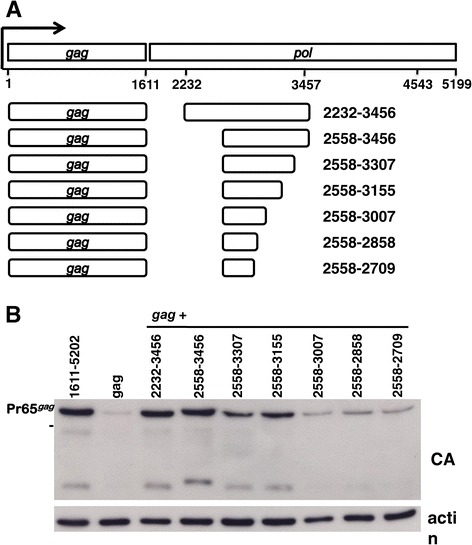
**Deletion analysis of the nucleotide 2232–3456** ***pol*** **fragment** ***.*** HEK293T cells were transfected with the indicated constructs derived from XMRV and harvested 48 hrs later for western blot. **(A)** Schematic showing truncation mutants generated from the XMRV *pol* fragment 2232–3456*.* **(B)** Cell lysate was probed with anti-CA antibody (upper panel) and anti-β-actin antibody (lowel panel).

To determine the shortest sequence within the first *pol* fragment that increased Gag protein levels, progressively larger deletion mutants were generated at the 5’ and 3’ termini of the *pol* 2232–3456 fragment. Based on initial mapping experiments in which the 3’ end was held fixed at nucleotide 3456, nucleotide 2558 was selected as the 5’ edge encoding the largest amino-terminal truncation that retained activity when cloned 3’ of gag coding sequence. Then, keeping the 5’ end fixed at 2558 progressively larger carboxy-terminal deletions were tested. The six resulting truncation mutants (Figure 4A) were cloned 3’ of *gag,* as described above. Each expression plasmid was transfected into 293 T cells alongside *gag* alone, *gag* 1611–5202 (bearing the complete *pol* sequence), and *gag* 2232–3456. Gag protein levels were determined in cell lysates with anti-CA antibody. Some decrease in Gag protein level was observed when the C-terminus was deleted beyond nucleotide 3456, although significant activity was still retained by the 597 nucleotide *pol* fragment 2232–3155 (Figure 4B).

### Mapping *pol* fragment 4543–5199

**Figure 5:**
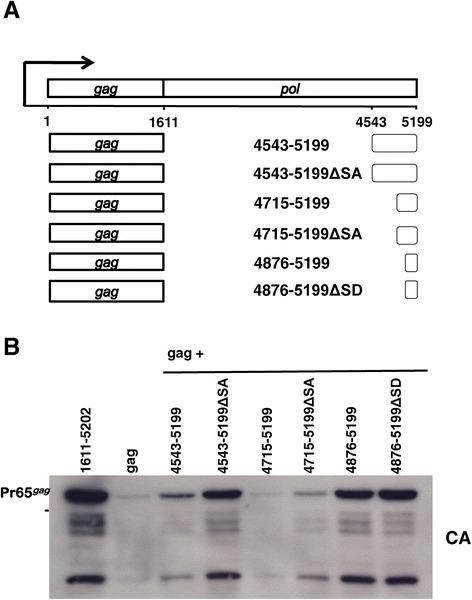
**Deletion mapping of** ***pol*** **fragment 4543–5199** ***.*** HEK293T cells were transfected with the indicated XMRV constructs, and harvested 48 hrs later. **(A)** Schematic of the truncation of XMRV *pol* fragment 4543–5199. Within this fragment is a known splice acceptor site (SA) and a potential splice donor (SD) site. Single point mutation within the AG sequence of the SA site and within the GU sequence of the SD site are shown. **(B)** Cell lysate was probed with anti-CA antibody (upper panel).

To determine the shortest sequence within the second *pol* fragment that increased Gag protein levels, three amino-terminal and three carboxy-terminal truncation mutants within *pol* fragment 4543–5199 were tested. Based on the activity of these, *pol* mutants 4876–5199 and 4715–5199 were selected for further study (Figure 5A). *pol* fragment 4715–5199 encompasses a known splice acceptor site [24-26] and a potential SD site [27,28]. The smaller fragment, 4876–5199, lacks the SA site but retains the putative SD site. Each fragment, either with the splice sites intact or with the sites mutated, was cloned 3’ of *gag* coding sequence and used to transfect 293 T cells (Figure 5A). Cell lysates were analyzed 48 hrs later in a western blot with anti-CA antibody (Figure 5B), and gel loading was normalized with a western blot for β-actin. The smaller truncation mutant, *pol* 4876–5199, that retains only 323 nucleotides of *pol* sequence, had full activity. Interestingly, mutation of either the SA or SD site by a single point mutation increased Gag production by either *pol* fragment (Figure 5B).

### XMRV *pol* only modestly increases total and cytoplasmic *gag* mRNA levels

**Figure 6:**
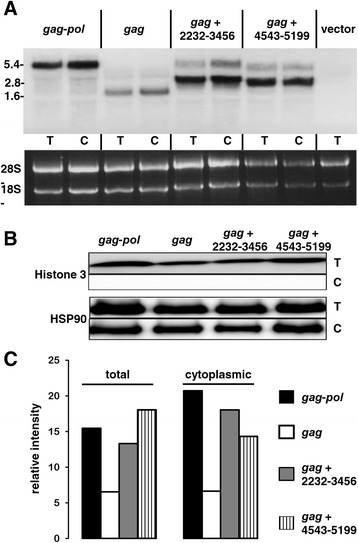
**The XMRV** ***pol*** **sequence contributes a modest extent to steady-state level and nucleocytoplasmic export of** ***gag*** **mRNA.** HEK293T cells were transfected with the indicated XMRV constructs. 48 hrs later, RNA from total (T) and cytoplasmic (C) fractions was collected. **(A)** XMRV mRNA was detected by northern blot with a radiolabeled *gag* mRNA probe. **(B)** The total cellular and cytoplasmic fractions used in northern blots were subjected to western blot with anti-HSP90 and anti-Histone H3 antibodies to monitor potential contamination of cytoplasmic preparations with nuclear contents. **(C)** Phosphorimager quantification of XMRV mRNA. Quantification was accomplished by detecting the bands and subtracting the background for each lane. This experiment - from transfection to northern blot quantitation - was repeated on three occasions with comparable results (see text). A representative experiment is shown.

The mechanism by which *pol* increases Gag polyprotein synthesis was considered next. Given that *pol* translation was not required for this effect (Figure 2B), *pol* might act to stabilize *gag* mRNA, to promote nuclear export of *gag* mRNA, or to promote translation of *gag* mRNA. As a first step to determine which of these three mechanisms was responsible for the effect on Gag, RNA was isolated from 293 T cells 48 hrs after transfection of 293 T with expression plasmids for *gag*-*pol*, *gag* alone, or *gag* with either of the two *pol* fragments - 2232–3456 or 4543–5199 - that increased Gag protein levels (Figure 3B). Total RNA from whole cell or cytoplasmic fractions was probed in a northern blot using a 1.6 kB riboprobe generated from full-length *gag* template. The transfection and northern blot experiment was repeated three times and Figure 6A shows a representative experiment. Western blot with anti-histone 3 antibody confirmed that the cytoplasmic fractions were not contaminated with nuclear contents (Figure 6B). Bands of the expected size were observed for each of the four transfected expression plasmids (Figure 6A): *gag-pol* (5202 nucleotides), *gag* (1611 nucleotides), *gag* + 2232–3456 (2835 nucleotides), and *gag* + 4543–5199 (2267 nucleotides). No spliced RNAs were expected with these constructs and, accordingly, none were detected.

Quantitation of the bands on the northern blot using a phosphorimager revealed that, in the total RNA fraction, the signal was 2.33 to 2.65-fold higher for *gag-pol* than for *gag,* with a standard deviation of 0.18, when data for the three independent experiments was assessed. The cytoplasmic signal for *gag-pol* was 1.22 to 3.12-fold higher than for *gag*-only, with a standard deviation of 1.05 when data was combined for the three independent experiments (Figure 6C). The *pol* fragment 2232–3456 increased total RNA 2-fold and cytoplasmic *gag* mRNA levels three-fold compared to *gag*-only. *pol* fragment 4543–5199 increased total and cytoplasmic *gag* mRNA levels about two-fold compared to *gag*-only. These results demonstrate that *pol* has a small effect on the stability and the nuclear export to the cytoplasm of *gag* RNA. The magnitude of these effects is at least an order of magnitude too small to explain the much larger magnitude effect of *pol* on Gag protein production (Figure 1B). This suggests that there is an effect of *pol* RNA that occurs after nuclear export of the *gag* RNA.

### *pol* promotes the association of *gag* mRNA with polyribosomes

**Figure 7:**
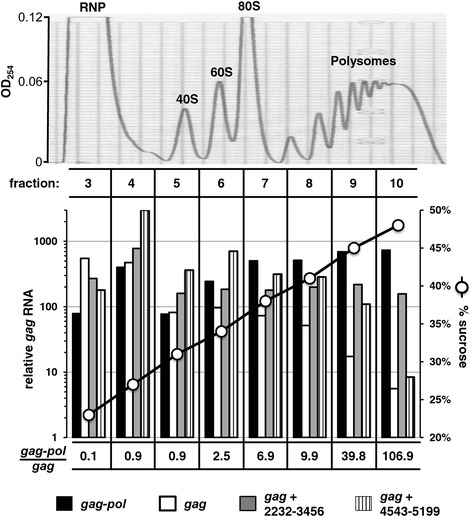
**Gammaretroviral** ***pol*** **promotes the association of** ***gag*** **mRNA with polyribosomes.** HEK293T cells were transfected with expression plasmids for *gag-pol*, *gag*, *gag* with *pol* fragment 2232–3456, or *gag* with *pol* fragment 4543–5199. 48 h post-transfection cell lysate was harvested and loaded onto a 15% to 55% linear sucrose gradient. After acceleration for 3 hrs at 210,000 × *g* RNA content across the gradient was assessed by reading absorbance at 254 nM (upper panel). The position of migration of the various ribosomal components and polysomes is indicated. Fractions were collected from the gradient (lower panel) and *gag* mRNA in fractions 3 to 10 was quantified by qRT-PCR relative to GAPDH as a control (left Y axis) and displayed against the percent sucrose (right Y axis). The ratio of *gag* RNA signal expressed from *gag-pol* plasmid versus the *gag*-alone alone plasmid is shown for each fraction. This experiment was repeated on three separate occasions, using comparable constructs from MLV and XMRV, and a representative experiment with MLV is shown.

Since the effect of *pol* on the cytoplasmic level of *gag* mRNA was too small to account for its >30-fold stimulation of Gag translation (Figure 2D), the effect of *pol* on *gag* mRNA association with polysomes was assessed. HEK293T cells were transfected with expression plasmids for *gag-pol*, *gag* alone, or *gag* with either of the two *pol* fragments, 2232–3456 or 4543–5199. 48 hrs later, cells were treated with cycloheximide to trap mRNA that was associated with ribosomes. Cells were lysed with mild, non-ionic detergent and nuclei were pelleted. The remaining cytoplasmic contents were loaded onto a 15% to 55% linear sucrose gradient and accelerated at 210,000 × *g* for 3 hrs. RNA content across the gradient was determined by reading absorbance at 254 nm and the typical profile of ribosome components, monosomes, and polysomes was observed (Figure 7, upper panel) Ten fractions were collected and the percent sucrose in each was determined by measuring the refractive index (Figure 7, lower panel, right vertical axis). Fractions one and two were discarded because the RNA content was too low for analysis. RNA was isolated from fractions 3 to 10 and the relative amount of *gag* RNA was determined by qRT-PCR. Results from a representative experiment are shown in Figure 7, lower panel, left vertical axis. The amount of *gag* mRNA associated with polyribosomes (fractions 8 to 10) was much higher when it was transcribed from the *gag-pol* plasmid than from the *gag* alone plasmid. Specifically, values from repeat experiments ranged from 9.9 to 38.5-fold higher for *gag-pol* than *gag* in fraction 8, 39.8 to 58.1-fold higher for fraction 9, and 106.9 to 130.5-fold higher for fraction 10. In contrast, signal ranged from 0.9 to 2.23-fold higher for ribonucleoprotein fraction 4. As compared with the *gag* alone plasmid, the plasmid containing *pol* fragment 2232–3456 increased the association of *gag* mRNA with polysomes 13-fold and the plasmid with *pol* fragment 4543–5199 increased it 6-fold. These results demonstrate that *pol* sequences promote the loading of *gag* mRNA onto polyribosomes with a magnitude that is sufficient to explain the effect of *pol* on Gag protein production.

### Translation of gammaretroviral *gag* RNA is stimulated by fusion to the MPMV CTE or by overexpression of NXF1/TAP and NXT/p15

**Figure 8:**
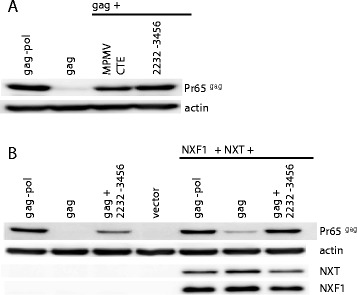
**MPMV CTE and NXF1/NXT promote gammaretroviral Gag protein production.** HEK293T cells were transfected with plasmids expressing *gag-pol*, *gag*, or *gag* with the 2232–3456 *pol* fragment. In addition, cells were transfected with a plasmid in which a single MPMV CTE was cloned downstream of *gag* **(A**), or cells were co-transfected with expression plasmids for NXF1 and NXT, as indicated in **(B)**. Cell lysate was harvested 48 hrs later and probed with anti-CA antibody (upper panel) or anti-β-actin antibody (lowel panel). Cell lysate from (B) was probed additionally with anti-NXF1 and anti-NXT antibody, as indicated. Similar results were obtained with identical constructs from MLV and XMRV. A representative experiment with MLV derived constructs is shown.

The stimulation of gammaretroviral Gag polyprotein synthesis by *cis*-acting *pol* RNA was reminiscent of the well-characterized MPMV CTE, a cis-acting RNA element that promotes nuclear export and translation of intron-containing MPMV mRNA [29-31]. To determine if the MPMV CTE would substitute for gammaretroviral *pol* to promote Gag protein synthesis, a single MPMV CTE was cloned downstream of the gammaretroviral *gag* open reading frame. When this plasmid was transfected into 293 cells the CTE increased Gag protein production up to the level observed with the *gag-pol* plasmid or the *gag* plasmid containing the 2232–3456 pol fragment (Figure 8A).

The MPMV CTE promotes translation of intron-containing RNAs by directly binding and recruiting NXF1/TAP, along with its cofactor NXT/p15 [31-33]. Previous studies showed that overexpression of NXF1 and NXT1 in 293 cells greatly boosted translation of HIV-1 *gag-pol*, but only when HIV-1 *gag-pol* was fused to the MPMV CTE [31]. The effect of NXF1/NXT overexpression on gammaretroviral Gag protein production was examined next. Plasmids encoding FLAG-tagged NXF1 and FLAG-tagged NXT were co-transfected with the plasmid expressing gammaretroviral *gag*-only or with the plasmid expressing *gag* fused to *pol* fragment 2232–3456. A slight increase in Gag protein production was observed with the gag only plasmid (Figure 8B). A greater increase in Gag protein production was observed when *gag* was fused to *pol* fragment 2232–3456. Taken together, these results suggest that gammaretroviral *pol* recruits NXF1/NXT to promote Gag protein production in in manner similar to that of the MPMV CTE.

### Gammaretroviral *pol* contains a CTE-like element that increases Gag protein production in an NXF1-dependent manner

**Figure 9:**
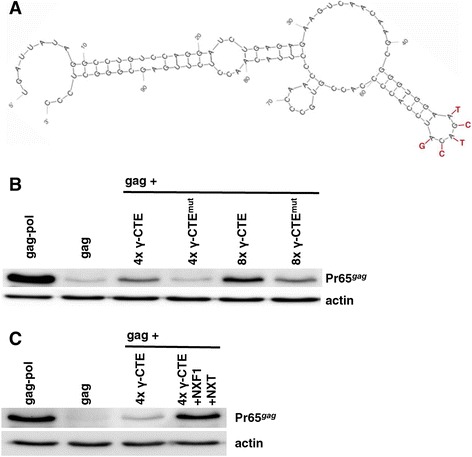
**Identification of a gammaretroviral CTE (**γ**-CTE) in** ***pol*** **. (A)** Secondary structure model of the minimal *pol* RNA fragment that stimulates Pr65^Gag^ production. This structure includes a loop with the NXF1-binding motif (AAGACA) found in the MPMV/MLV CTE (identical for both viruses). Nucleotides that were mutated to generate γ-CTE^mut^ (ATCGCT) are highlighted in red. In **(B)** and **(C)**, HEK293T cells were transfected with expression plasmids for *gag*, *gag-pol*, *gag* with 4 copies of either wild-type or mutant γ-CTE, NXF1, and NXT, as indicated. Cell lysate was harvested 48 hrs later and probed with anti-CA antibody and anti-β-actin antibody. Similar results were obtained with identical constructs from MLV and XMRV. A representative experiment with MLV derived constructs is shown.

Given the increased NXF1/NXT-responsiveness of gammaretroviral *Gag* protein production when *gag* was fused to *pol* 2232–3456, the *pol* fragment RNA was examined for primary and secondary structural elements that resemble the CTE of MPMV or the CTE located in intron 10 RNA of NXF1 itself [30]. Using m-fold [30,34] to scan *pol* sequence fragments of 100 nucleotides an RNA stem-loop (ΔG = −26.55 kcal/mol) was identified at *pol* nucleotides 2292–2390 (Figure 9A) that possessed an AAGACA loop characteristic of the MPMV and NXF1 CTEs that have been reported to bind and recruit NXF1 [30,35-39]. Little effect on Gag polyprotein synthesis was observed when one, two, or three tandem copies of this putative gammaretrovirus CTE (γ-CTE) were cloned downstream of gammaretrovirus *gag* (data not shown). Four tandem copies of the γ-CTE were associated with an obvious increase in Gag production and eight tandem copies increased Gag protein further, nearly to the same level as the complete *pol* sequence (Figure 9B).

Two experiments provided further evidence that the recruitment of NXF1 by the γ-CTE is relevant for stimulation of Gag polyprotein synthesis by *pol*. When the AAGACA motif in the γ-CTE was mutated to ATCGCG (γ-CTE^mut^), a mutation that was previously shown to disrupt NXF1 binding [37], the effect of the γ-CTE on Gag polyprotein production was attenuated (Figure 9B). Additionally, overexpression of NXF1 and NXT increased the effect of the γ-CTE on Gag polyprotein production (Figure 9C). Taken together, these results suggest that, at least in part, gammaretroviral *pol* increases Gag polyprotein synthesis by recruiting NXF1.

### The impact of NXF1 knockdown on Gag protein production

Since NXF1 overexpression increased Gag polyprotein production, the effect of NXF1 knockdown was examined next. Three NXF1 target sequences were engineered into the miR30 framework of a previously described lentiviral knockdown vector that, additionally, confers resistance to puromycin [40,41]. HEK293 cells transduced with either of the three NXF1 knockdown vectors were eliminated from the culture upon addition of puromycin. The magnitude of this apparent toxicity correlated with the efficiency of the knockdown, as assessed by western blot for NXF1. Cells transduced with a control vector targeting luciferase propagated normally in the presence of puromycin.

**Figure 10:**
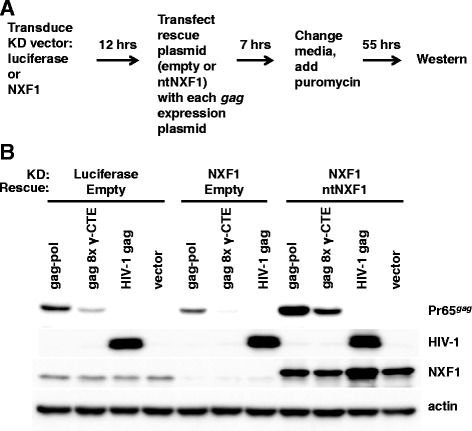
**The effect of NXF1 KD on Pr65** ^**Gag**^ **protein production. (A)** Diagram showing protocol for short-term selection of cells with NXF1 KD. HEK293 cells were transduced with lentiviral vectors bearing miR30 frameworks targeting either luciferase or NXF1. Then, cells were transfected with the indicated plasmids, and finally selected in pools with puromycin. **(B)** 55 hrs after addition of puromycin cell lysate was probed by western blot with anti-gammaretrovirus CA antibody, anti-HIV-1 CA antibody, anti-NXF1 antibody, or anti-β-actin antibody, as indicated. Similar results were obtained with identical constructs from MLV and XMRV. A representative experiment with MLV derived constructs is shown.

Given that NXF1 is essential in HEK293 cells, a short-term protocol was developed to test the effect of NXF1 knockdown on gammaretroviral Gag polyprotein production (Figure 10A). Cells were transduced with an NXF1 knockdown vector targeting the 3’UTR, or with a control vector targeting luciferase. 12 hrs later, the transduced cells were transfected with either an NXF1 open reading frame expression plasmid that is not targeted by the knockdown vector (ntNXF1), or with an empty expression plasmid. Simultaneously, the same cells were co-transfected with expression plasmids encoding either gammaretroviral *gag-pol* or gammaretroviral *gag* fused to eight copies of the γ-CTE. A Rev-dependent HIV-1 *gag*-*pol* expression plasmid, that also expresses *rev* and bears the *cis*-acting RRE, served as a control for potential non-specific effects resulting from NXF1 knockdown since HIV-1 structural protein production is CRM1-dependent [11] and should not require NXF1 [31]. 7 hrs after transfection the media was replaced and puromycin was added to 8 μg/ml. 55 hrs later, the puromycin-selected cells were assayed by western blot for NXF1, gammaretroviral Gag, and actin protein levels (Figure 10A).

As compared with Gag polyprotein levels in the control luciferase knockdown cells, Gag polyprotein levels in the NXF1 knockdown cells were reduced, whether the Gag was encoded by the *gag*-*pol* plasmid or by the plasmid in which *gag* was fused to the γ-CTE (Figure 10B). Gag polyprotein levels produced by the *gag* alone plasmid were too low to test for reduction by NXF1 knockdown. When NXF1 levels were restored to supra-normal levels in the NXF1 knockdown cells by introduction of a non-targetable NXF1 expression plasmid, Gag polyprotein production was increased compared to the control cells (Figure 10B). NXF1 knockdown had no effect on the CRM1-dependent HIV-1 structural protein (Figure 10B), indicating that the reduction of gammaretroviral Gag polyprotein production was specific and not due to non-specific toxicity. These results indicate that gammaretroviral *pol* sequences promote Gag polyprotein production in an NXF1-dependent fashion.

### SRp20 promotes gammaretrovirus Gag production by recruiting NXF1/NXT1

**Figure 11:**
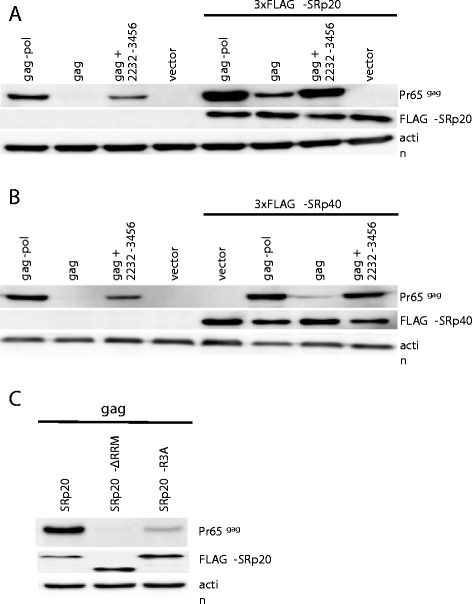
**SRp20 promotes Pr65** ^**Gag**^ **production in an NXF1-dependent manner.** HEK293T cells were co-transfected with expression vectors for either *gag-pol*, *gag*, or *gag* with the 2232–3456 *pol* fragment. Each of these was co-transfected with either FLAG-tagged SRp20 **(A)** or FLAG-tagged SRp40 **(B)**. In **(C)**, cells were transfected with an expression plasmid for *gag* and either wild-type or mutant SRp20. One mutant, SRp20ΔRRM, lacks the RNA recognition motif. The other mutant, SRp20R3A, disrupts binding to NXF1. 48 hrs later cell lysate was probed with anti-CA antibody, anti-Flag antibody, and anti-β-actin antibody, as indicated. Similar results were obtained with identical constructs from MLV and XMRV. A representative experiment with MLV derived constructs is shown.

SR proteins promote nuclear export and translation of mRNAs in eukaryotic cells [42-44]. Among the SR proteins, SRp20 shuttles between the nucleus and the cytoplasm and recruits NXF1/NXT to mRNAs [45-48]. Given the importance of NXF1/NXT for gammaretroviral Gag polyprotein production the effect of SRp20 was examined next. When cells were co-transfected with the *gag*-only expression plasmid and an SRp20 expression plasmid, Gag polyprotein production was increased to the level observed with the *gag-pol* plasmid in the absence of SRp20 overexpression (Figure 11A). Significant further increase in Gag polyprotein production was also observed when SRp20 was overexpressed with the *gag-pol* plasmid or with the plasmid bearing *gag* along with the *pol* fragment 2232–3456 (Figure 11A). Overexpression of SRp20 in the same system had no effect on protein production by an HIV-1 *gag* expression plasmid (data not shown and reference 43). To determine if the effects of SRp20 overexpression were specific to this SR protein family member, an identical expression construct was generated for SRp40, an SR protein that does not shuttle between the nucleus and the cytoplasm and does not recruit NXF1/NXT to mRNAs [49,50]. In contrast to what was observed with SRp20 overexpression, no significant increase in gammaretroviral Gag polyprotein production was observed with overexpression of SRp40 (Figure 11B), a condition that stimulated HIV-1 Gag production (43).

SRp20 has two main structural domains, an RNA recognition motif (RRM) and a carboxy-terminal RS domain. Based on previous work [51], two SRp20 mutants were generated. The first mutant, SRp20ΔRRM, lacks the RRM. This mutant retains the ability to bind NXF1 but is unable to bind RNA. When overexpressed, SRp20ΔRRM caused no detectable increase in Gag protein production (Figure 11C). The second mutant, SRp20R3A, has three arginines at amino acid positions 256, 262, and 268 in the RS domain all mutated to alanine. SRp20R3A retains the ability to bind RNA but is unable to recruit NXF1 [51]. When SRp20R3A was co-transfected with the gammaretroviral *gag* expression plasmid, no significant increase in Gag protein production was observed (Figure 11C). Thus, both RNA binding via the RRM, and recruitment of NXF1 via the RS domain, are required for SRp20 stimulation of Gag protein production.

### SRp20 increases gammaretroviral *gag* mRNA association with polysomes in an NXF1-dependent manner

**Figure 12:**
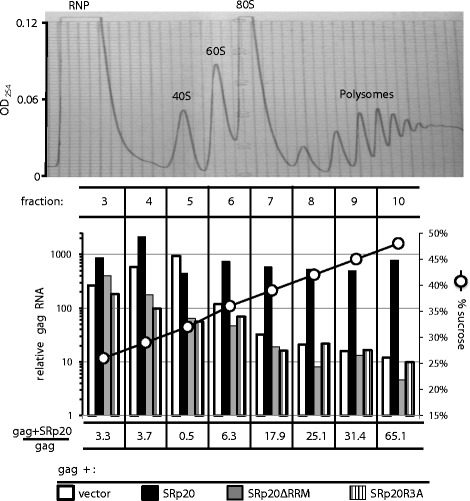
**SRp20 promotes** ***gag*** **mRNA association with polysomes in an NXF1-dependent manner.** HEK293T cells were transfected with expression plasmids for *gag* and either SRp20 wild-type, SRp20ΔRRM, or SRp20R3A. 48 h post-transfection cell lysate was loaded onto a 15% to 55% linear sucrose gradient. After acceleration for 3 hrs at 210,000 × *g*, RNA content across the gradient was assessed by reading absorbance at 254 nM (upper panel). The position of migration of the various ribosomal components and polysomes is indicated. Fractions were collected from the gradient (lower panel) and relative *gag* mRNA in fractions 3 to 10 was quantified by qRT-PCR using GAPDH as a control (left Y axis) and displayed against the percent sucrose (right Y axis). The ratio of *gag* RNA signal expressed in the presence of SRp20 wild-type, versus the *gag* RNA signal in the absence of SRp20 plasmid is shown for each fraction. This experiment was repeated on three separate occasions with the same result. Similar results were obtained with identical constructs from MLV and XMRV. A representative experiment with MLV-derived constructs is shown.

As was demonstrated with the gammaretroviral *pol* sequences (Figure 6), the effect of SRp20 overexpression on the levels of gammaretroviral *gag* mRNA was much smaller than the increase in Gag polyprotein levels (data not shown). Therefore, as was done with the *pol* sequences (Figure 7), the effect of SRp20 overexpression on *gag* mRNA association with polysomes was assessed. HEK293T cells were co-transfected with the *gag* expression plasmid and either the SRp20 wild-type, SRp20ΔRRM, SRp20R3A, or empty expression plasmids. 48 hrs later, cells were treated with cycloheximide, lysed with non-ionic detergent, and cytoplasmic contents were loaded onto a 15% to 55% linear sucrose gradient for polysome analysis as described above. Fractions were collected, RNA was isolated, and *gag* RNA in each fraction was quantitated by qRT-PCR. SRp20 increased the association of *gag* with polyribosomes about 30-fold (one representative of 3 experiments is shown in Figure 12). Neither, SRp20ΔRRM nor SRp20R3A caused any detectable shift of *gag* mRNA from monosomes to polysomes (Figure 12). These results demonstrate that both RNA binding activity and recruitment of NXF1 are required for the stimulation of Gag polyprotein production by SRp20.

## Discussion

The genomic RNA of all retroviruses, as well as the mRNA that is translated to produce the *gag* and *pol* gene products, resembles unspliced pre-mRNA in that it possesses splice donor and acceptor sites. Different classes of retroviruses have evolved unique mechanisms for exporting these unspliced mRNAs out of the nucleus in a manner that ensures efficient loading onto polysomes for translation. How unspliced gammaretrovirus mRNA is exported and translated has not been extensively investigated. Among retroviruses, gammaretroviruses are relatively simple in structure, possessing only three genes, *gag*, *pol*, and *env*, and lacking accessory proteins such as HIV-1 Rev that act *in trans* to promote the nuclear export of unspliced mRNA. It therefore seemed likely that gammaretroviruses would behave similar to the betaretrovirus MPMV that uses *cis*-acting elements in the viral RNA to recruit cellular factors that promote nuclear export and translation of the mRNA.

The studies here focused on Gag polyprotein translation directed by the unspliced *gag* mRNA from either of two gammaretroviruses, XMRV or Moloney-MLV. In isolation, the *gag* open reading frame was incapable of directing Gag polyprotein translation, even when *gag* transcription was driven by the potent CMV immediate early promoter (Figure 1). The primary sequence of the *gag* open reading frame was responsible for the block to translation since introduction of a large number of silent mutations permitted production of copious amounts of Gag polyprotein (Figure 2).

It was then demonstrated here that translation of gammaretroviral *gag* mRNA relies upon *cis*-acting sequences within *pol* (Figure 2). The location of these gammaretroviral elements is an interesting contrast to the well-characterized betaretroviral CTE which is located at the 3’ end of the MPMV genome. Analysis of gammaretroviral *pol* deletion mutations (Figure 3B) showed that two different fragments, one from nucleotides 2232–3456 (Figure 4B) and the other from nucleotides 4543–5199 (Figure 5B), contribute to *gag* mRNA translation. Though each fragment alone was quite potent at increasing Gag polyprotein levels, neither was equivalent to the complete *pol* sequence, indicating that the effect of the two fragments is additive. Our *pol* mapping results are in agreement with a recent report that also identified the 3’ end of *pol* as important for gammaretroviral Gag translation [52].

As determined by northern blot and by RT-PCR, each of the two *pol* sequences increased the steady-state level of total *gag* mRNA, as well as the level of cytoplasmic *gag* mRNA (Figure 6). But the magnitude of these effects was only a few fold, much smaller than the greater than 30-fold increase in Gag polyprotein translation associated with *pol* (Figure 1B). Rather, the main effect of the *pol* sequences was to promote loading of the *gag* mRNA onto polysomes, as determined by RT-PCR of the fractions obtained from a polysome gradient (Figure 7). In contrast to the results reported here, another group reported that the major effect of gammaretroviral *pol* is to promote nuclear export of either spliced or unspliced transcripts [52].

The biochemical characterization of the murine gammaretroviral elements described here, and elsewhere [52], was performed using human cells, and it is possible that their relative importance for RNA stability, transport, or polysome loading would be somewhat different in murine cells. That being said, though XMRV is a recombinant of two mouse retroviruses this virus was selected for replication in a human prostrate cancer cell line [53] and so the use of a human cell line for the XMRV biochemical studies described here is not unreasonable.

Clues to the mechanism of action of the gammaretroviral *pol* sequences were also obtained by considering the MPMV CTE, which was able to substitute for the *pol* sequences to promote gammaretroviral Gag polyprotein production (Figure 8A). It had been shown previously that a single copy of the MPMV CTE permits production of HIV-1 structural proteins in the absence of Rev [14], though in some contexts this required four tandem CTE repeats, or overexpression of the shuttling SR protein 9G8 or the export receptor NXF1/NXT [29,30,42,43]. A single MPMV CTE was sufficient to promote gammaretroviral Gag production (Figure 8).

Given the similarity with MPMV, an RNA stem-loop with properties resembling the MPMV CTE was sought within gammaretroviral *pol*. A 100 nucleotide *pol* fragment was identified (Figure 9A) that bears a AAGACA motif like that previously shown to bind to NXF1 [33]. Repeats of these stem-loops increased Pr65^Gag^ production in a manner that was dependent on the AAGACA motif and that synergized with overexpression of NXF1/NXT (Figure 9B and C). Conversely, NXF1 knockdown decreased gammaretroviral Gag polyprotein production (Figure 10). Though NXF1 knockdown was toxic to the cells, and clearly limited the magnitude of the effect that was possible to detect, there was no effect on Rev-dependent synthesis of HIV-1 structural protein (Figure 10). These results suggest that gammaretroviral *pol* sequences recruit NXF1 to promote Gag polyprotein synthesis. Though direct binding between NXF1 and *pol* sequences was not demonstrated here, such an interaction was recently reported [52].

Gammaretroviral *gag* behaves like an intronless gene. 5% of eukaryotic genes naturally lack introns [54]. The intronless histone H2A mRNA encodes a *cis*-acting element that recruits SR proteins [45,46,55] and subsequently NXF1/NXT, to promote nuclear export and translation [56-58]. SRp20 in particular recruits NXF1/NXT with which it forms a ternary complex [59]. SR proteins are involved in post-transcriptional processing of mRNA [50,60], and contribute to the coupling of splicing, nuclear export and translation initiation [45,46]. In some cases, NXF1/NXT associates with the NPC and promotes nuclear export of mRNAs by binding to adaptor proteins rather than by direct RNA binding [61,62].

**Figure 13:**
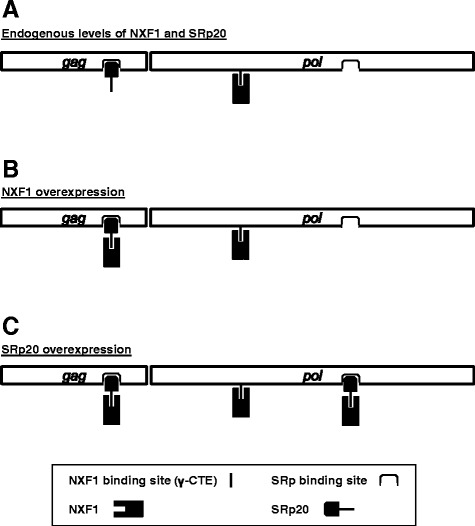
**Model for the post-transcriptional regulation of gammaretroviral** ***gag*** **mRNA by NXF1 and SRp20.** Translation of gammaretroviral Gag polyprotein requires recruitment of NXF1 to the *gag* mRNA. Recruitment of NXF1 might be direct, as it is with the MPMV CTE and appears to be with the γ-CTE located in *pol*, or indirect, via binding SRp20. **(A)** When NXF1 and SRp20 proteins are present at endogenous levels, direct binding of NXF1 to the γ-CTE in *pol* appears to be essential for Gag translation. **(B)** When NXF1 is overexpressed, some Gag protein is produced by *gag* alone, perhaps due to indirect NXF1 recruitment by SRp20. **(C)** When SRp20 is overexpressed, significant increase in Gag protein is observed with either *gag* alone or with *gag-pol*.

Further evidence for the importance of NXF1 for Gag polyprotein production was provided here by experiments examining the effect of overexpression of serine/arginine (SR)-rich proteins. Overexpression of SRp20 drove an increased production of Gag polyprotein by the *gag-pol* construct (Figure 11A). This was associated with a large increase in polysome loading of gag mRNA (Figure 12). Hargous and coworkers had identified three arginines within the SRp20 RS repeat that are critical for NXF1 binding [51]. Mutation of these residues, as well deletion of the RNA-binding RRM motif, disrupted the effect of SRp20 overexpression on Gag protein production (Figure 12). Thus, both the RNA-binding and NXF1-recruitment activities of SRp20 were essential for the promotion of Gag polyprotein synthesis. These experiments suggest that *pol* not only possesses motifs that directly recruit NXF1 but that it also recruits adaptor proteins like SRp20 which indirectly recruit NXF1 (Figure 13).

Overexpression of NXF1/NXT increases the nucleocytoplasmic export of MPVM CTE-containing mRNAs by about 2-fold [31] and overexpression of mRNAs containing a CTE can block the nucleocytoplasmic export of cellular mRNAs [16,63]. Sites on the RNA for direct NXF1-binding therefore seem to be saturable and therefore limiting (Figure 13). Overexpression of SRp20 appears to recruit more NXF1 to mRNA than NXF1 overexpression can provide, perhaps because the NXF1 binding sites on mRNA are less abundant than potential SRp20 binding sites (Figure 13). Gammaretroviral *gag* mRNA itself may possess SRp20 binding sites since SRp20 overexpression stimulated Gag polyprotein production by *gag* in the absence of *pol* sequences (Figures 11 and 12). According to this model, the increased SRp20 protein that is bound to *gag* mRNA then would recruit more NXF1, increasing the polysome loading of *gag* mRNA (Figures 12 and 13).

It has been shown that shuttling proteins like ASF/SF2 and 9G8 associate with polyribosomes [42,44,64]. The non-shuttling SR proteins like SRp40 only accompany the mRNA until the NPC and dissociate with their release into the cytoplasm. Consequently, SRp40 might not support the association of mRNA with polyribosomes and subsequent translation initiation. In fact, the non-shuttling SR protein, SRp40, did not promote Gag polyprotein translation like SRp20 (Figure 11B). Coyle and coworkers showed that the CTE element of MPMV mediates efficient export of reporter mRNA in HEK293T cells [29]. In contrast to the experiments here, the overexpression of shuttling SR proteins had no effect on the nucleocytoplasmic export of CTE-containing mRNAs [42].

Other host factors were sought here that might contribute to gammaretroviral protein production. One of the best characterized mRNAs in mammalian cells is the β-actin mRNA. Proper localization of β-actin mRNA within the cell mediates cell migration during embryogenesis, differentiation and possibly carcinogenesis [65-69]. The zip code binding protein 1 (Zbp1) prevents premature translation of the β-actin mRNA during transport. Once the β-actin mRNA reaches its final destination Zbp1 is released from the mRNA and translation can occur [70]. To determine if translational repression by the human homologue of Zbp1 (known as IMP1 or IGF2BP1) inhibits Gag polyprotein translation Imp1 was efficiently knocked down in HEK293T cells. No effect of IMP1 knockdown on Gag polyprotein production was observed (data not shown).

Tpr is a 254 kDa protein localized to the nuclear basket of the nuclear pore complex [71,72]. Tpr suppresses expression of unspliced and intron-containing mRNA by inhibiting the export of CTE-containing RNA but not Rev-dependent mRNA exported via the CRM1-pathway [73]. Since gammaretroviral *pol* seems to behave like a CTE the effect of Tpr knockdown was examined next. No significant change in Gag polyprotein levels was observed with Tpr knockdown in HEK293T cells (data not shown).

## Conclusions

Little has been reported concerning post-transcriptional regulation of gammaretroviral genes. Here it was shown that Gag polyprotein synthesis is strictly dependent upon a 100 nucleotide element in *pol* RNA that resembles the CTE of the betaretrovirus MPMV. Like the MPMV CTE, the γ-CTE described here appears to recruit the cellular factor NXF1. Overexpression of either NXF1/NXT or of SRp20 serves to increase gammaretroviral Gag polyprotein production. While γ-CTE contributes to *gag* mRNA stability and export to the cytoplasm, it’s major effect is to promote polysome loading of the *gag* mRNA.

## Methods

### Cell lines, tissue culture, and drugs

HEK293T cells were grown in Dulbecco’s modified Eagle medium (D-MEM) (Invitrogen) supplemented with 10% fetal bovine serum (FBS). Puromycin was used up to a concentration of 8 μg/ml.

### Plasmids

The background of all the designed plasmids is pcDNA3.1. Xenotropic MuLV-related virus VP62 (XMRV, accession number NC_007815) *gag-pol* was generated by polymerase chain rection (PCR) in three fragments. The first fragment was amplified with primers X01f and X01r (Table 1) and cloned with NheI and KpnI into pcDNA3.1(+). The second fragment was amplified with primers X02f and X02r and cloned with KpnI and XhoI into pBS SK(+). The third fragment was amplified with primers X03f and X03r and cloned with XhoI and NotI into pBS-SK(+). Subsequently the second and the third fragment were subcloned KpnI-NotI into pcDNA3.1(+) that already contained the first fragment NheI-KpnI.

**Table 1:** **Oligonucleotides used in this study**

**Primer name**	**Primer sequence**
X01f	5’-CACACAGCTAGCATCATGGGACAGACCGTAACTACCCCTCTG-3’
X01r	5’-GCGAAGGCATAGCGGCTATCAGTGT-3’
X02f	5’-ATCCACTTCGAGGGATCAGGAGCTC-3’
X02r	5’-GCGAAGGCATAGCGGCTATCAGTGT-3’
X03f	5’-AACTGGGACCTTGGCGTCGGC-3’
X03r	5’-GTGTGTGCGGCCGCTCAGGGGGCCCCACGGGT-3’
M01f	5’-CACACAGCTAGCATGGGCCAGACTGTTACCACTCCC-3’
M01r	5’-CTGGGCGCTCGAGGGGAAAAG-3’
M02f	5’-CTTTTCCCCTCGAGCGCCCAG-3’
M02r	5’-CTTCGGCCAGGATATCAAGGCAGTTG-3’
M03f	5’-CAACTGCCTTGATATCCTGGCCGAAG-3’
M03r	5’-GTGTGTGCGGCCGCTTAGGGGGCCCCACGGGTTAATC-3’
M04f	5’-CACACAGCGGCCGCCTGCCAGTCCCCCTGG-3’
M04r	5’-GTGTGTCTCGAGGATATCAAGGCAGTTGTGTTGCAGCC-3’
M05f	5’-CACACACTCGAGCTGCCAGTCCCCCTGG-3’
M05r	5’-GTGTGTTCTAGAGATATCAAGGCAGTTGTGTTGCAGCC-3’
X04f	5’-CACACAGGTACCCTGCCAGTCCCCCTGG-3’
X04r	5’-GTGTGTGAATTCGCAGTCATGGGGGGCTTCCTTTTC-3’
X05f	5’-CACACAGCGGCCGCCTGCCAGTCCCCCTGG-3’
X05r	5’-GTGTGTCTCGAGGCAGTCATGGGGGGCTTCCTTTTC-3’
X06f	5’-CACACACTCGAGCTGCCAGTCCCCCTGG-3’
X06r	5’-GTGTGTTCTAGAGCAGTCATGGGGGGCTTCCTTTTC-3’
X07f	5’-CACACAGGTACCATCTTGGCTGAGACGCACGGAACC-3’
X07r	5’-GTGTGTGAATTCTCAGGGGGCCCCACGGGTTA-3’
X08f	5’-CACACAGGTACCCCACCCTGTTTGATGAGGCACTGC-3’
X09r	5’-GTGTGTGAATTCGTTTGACCAGTGCTTCTACCGCATGG-3’
X10r	5’-GTGTGTGAATTCAGGTAGGCCACAGGCCGACGC-3’
X11r	5’-GTGTGTGAATTCCTTGATAGGCCTTTTGCTGGTCTGGG-3’
X12r	5’-GTGTGTGAATTCGTCTTCGGAGTGGGCTGCCCC-3’
X13r	5’-GTGTGTGAATTCGGTTTGTAATAGGGCCCGAGTACCTCG-3’
X14f	5’-CACACAGCGGCCGCGGAGGTCAGGGTCAGGAGCCCC-3’
X14r	5’-GTGTGTCTCGAGGAATTCGGTACCAGTATTCCCTGGTCCAACAGCC-3’
X15r	5’-GTGTGTGCGGCCGCTCAGTCACCTAAGGTCAGGAGGGAGGTCTG-3’
T01f	5’-CACACATCTAGAGCCACCATGGCGGACGAGGGGAAGTCG-3’
T01r	5’-GTGTGTGCGGCCGCTCACTTCATGAATGCCACTTCTGGGATC-3’
T02f	5’-CACACAGCTAGCGCCACCATGAGAAAATACAGAAGCCACTGGTCTCAG-3’
T02r	5’-GTGTGTGCGGCCGCTTAACTACTAGACCAATCTTGAAAACGGAAGC-3’
T03f	5’-CACACAGCTAGCGCCACCATGCATCGTGATTCCTGTCCATTGG-3’
T03r	5’- GTGTGTGCGGCCGCCTATTTCCTTTCATTTGACCTAGATCGACTACG-3’
T04f	5’- CACACAGCTAGCGAAAAAAGAAGTAGAAATCGTGGCCCAC-3’
T05f	5’- GAACTGTCGAATGGTGAAAAAGAAAGTGAAAATGAAGGCCCACCTCCCT
	CTTGGGG-3’
T05r	5’- CCCCAAGAGGGAGGTGGGCCTTCATTTTCACTTTCTTTTTCACCATTCGA
	CAGTTC-3’
T06f	5’- CACACAGCTAGCGCCACCATGAGTGGCTGTCGGGTATTCATCG-3’
T06r	5’- GTGTGTGCGGCCGCTTAATTGCCACTGTCAACTGATCTGGACC-3’
X18f	5’-GTCAACAAGCGGGTGGATCGCGTCCACCCCACCGTGCC-3’
X18r	5’-GGCACGGTGGGGTGGACGCGATCCACCCGCTTGTTGAC-3’
X19f	5’-GTCACCTTCCTGGTAAACACTGGCGCCCAACACTCCGTG-3’
X19r	5’-CACGGAGTGTTGGGCGCCAGTGTTTACCAGGAAGGTGAC-3’
X20f	5’-CACACAGAATTCCCTGCCTTCGCCTCCCAGGTAAG-3’
X20r	5’-GTGTGTCTCGAGGGGGCCCCACGGGTTAATCTTATC-3’
X21f	5’-AGCTCACTTACACGCCCTCCAAGCAGT-3’
X21r	5’-ACTGCTTGGAGGGCGTGTAAGTGAGCT-3’
X22f	5’-CTTCGCCTCCCAGCTAAGTCAGTCAG-3’
X22r	5’-CTGACTGACTTAGCTGGGAGGCGAAG-3’
X26f	5’-CACACAGAATTCGGAAAGGACCCTACACCGTCCTGC-3’
X27f	5’-CACACAGAATTCCCCTCCAAGCAGTACAACAAGAGGTCTG-3’
Mir30f	5'-AAGGCTCGAGAAGGTATATTGCTGTTGACAGTGAG-3'
Mir30r	5'-AGCCCCTTGAATTCCGAGGCAGTAGGCA-3'
NXF1 − + KD	5'-TGCTGTTGACAGTGAGCGACGACGTGCTTTGCTGTATAAATAGTGAAGCCAC
	AGATGTATTTATACAGCAAAGCACGTCGCTGCCTACTGCCTCGGA-3’
NXF1-2 KD	5’- TGCTGTTGACAGTGAGCGCACCCTGAGGATCATTGAAGAGTAGTGAAGCCA
	CAGATGTACTCTTCAATGATCCTCAGGGTATGCCTACTGCCTCGGA-3';
NXF1-3	5'-TGCTGTTGACAGTGAGCGATGCCAGGAAGCCAAAGCTTACTAGTGAAGCCA
KD	CAGATGTAGTAAGCTTTGGCTTCCTGGCACTGCCTACTGCCTCGGA-3'
Codon optimized XMRV *gag*	5’-ATGGGCCAGACCGTGACCACCCCCCTGAGCCTGACCCTGCAGCATTGGGG
	CGACGTGCAGCGGATCGCCAGCAACCAGAGCGTGGACGTGAAAAAGCGGAGATGGGTCACCTTCTGCAGCGCCGAGTGGCCCACCTTCAACGTGGGCTGGCCCCAGGACGGCACCTTCAATCTGGGCGTGATCAGCCAGGTCAAAAGCCGGGTGTTCTGCCCTGGCCCCCACGGACACCCTGACCAGGTGCCCTACATCGTGACCTGGGAGGCCCTGGCCTACGACCCTCCACCCTGGGTCAAGCCCTTCGTGTCCCCTAAGCCCCCACCCCTGCCTACAGCTCCAGTGCTGCCTCCTGGCCCTAGCGCCCAGCCTCCTAGCAGAAGCGCCCTGTACCCCGCCCTGACCCCATCCATCAAGAGCAAGCCCCCCAAGCCTCAGGTGCTGCCCGATTCTGGCGGCCCTCTGATCGACCTGCTGACCGAGGACCCCCCTCCATATGGCGCCCAGCCAAGCAGCAGCGCCAGAGAGAACAACGAGGAAGAGGCCGCCACCACCAGCGAGGTGTCCCCACCTAGCCCTATGGTGTCCCGGCTGCGGGGAAGAAGAGATCCTCCTGCCGCCGACAGCACCACCAGCCAGGCCTTCCCACTGAGAATGGGCGGCGACGGCCAGCTGCAGTACTGGCCTTTCAGCAGCAGCGACCTGTACAACTGGAAGAACAACAACCCCAGCTTCAGCGAGGACCCTGGCAAGCTGACCGCCCTGATCGAGAGCGTGCTGATCACCCACCAGCCCACCTGGGACGACTGCCAGCAGCTCCTGGGCACCCTGCTGACAGGCGAAGAGAAGCAGCGGGTGCTGCTGGAAGCCAGAAAGGCCGTGCGGGGCAACGACGGCAGACCTACCCAGCTGCCCAACGAAGTGAACGCCGCCTTCCCCCTGGAACGGCCCGACTGGGACTACACCACCACCGAGGGCCGGAACCACCTGGTGCTGTACAGACAGCTGCTGCTGGCTGGCCTGCAGAATGCCGGCAGAAGCCCCACCAACCTGGCCAAAGTGAAGGGCATCACCCAGGGCCCCAACGAGAGCCCCAGCGCCTTCCTGGAAAGACTGAAAGAGGCCTACCGGCGGTACACCCCCTACGATCCTGAGGACCCTGGCCAAGAAACAAACGTGTCCATGAGCTTCATCTGGCAGAGCGCCCCTGACATCGGCCGGAAGCTGGAACGGCTGGAAGATCTGAAGTCCAAGACCCTGGGGGACCTCGTGCGCGAGGCCGAGAAGATCTTCAACAAGAGAGAGACACCCGAGGAACGGGAAGAGAGAATCCGGCGCGAGATCGAGGAAAAAGAGGAACGGCGCAGAGCCGAGGACGAGCAGAGAGAGCGCGAGAGAGACAGACGGCGGCACAGAGAGATGAGCAAGCTGCTGGCCACCGTGGTCATCGGCCAGCGGCAGGATAGACAGGGCGGCGAGAGAAGAAGGCCCCAGCTGGACAAGGACCAGTGCGCCTACTGCAAAGAGAAGGGCCACTGGGCCAAGGACTGCCCCAAGAAGCCCAGAGGACCTAGGGGCCCTAGACCTCAGACCAGCCTGCTGACACTGGGCGATTGA-3’

XMRV *gag-pol*^*fs*^ has a mutation at the 5’ end of *pol* sequence that puts the pol sequence out of frame with respect to *gag*. BstEII digestion and subsequent fill-in of the 5’ overhang by T4 DNA polymerase generated a frameshift mutation at the 5’ end of the *pol* sequence with stop codons downstream that block *pol* translation.

XMRV *gag* was amplified with primers X01f and X15r as an NheI-NotI fragment. The codon optimized XMRV *gag* open reading frame was synthesized by GENEART AG (Regensburg, Germany) and inserted into the Nhe1 and Not1 sites of pcDNA3.1. The codon optimized *gag* sequence is shown in Table 1. It has 398 silent nucleotide changes with respect to the original gag sequence. The GC content of the codon optimized *gag* is 64%, as compared to 55% in the original.

The mapping of XMRV *pol* sequence required a construct that contained XMRV*gag* plus *pol* sequences downstream. The 5’ end of *pol* was amplified with primers X14f and X14r as a linker, and cloned NotI-XhoI downstream of XMRV *gag*. Additional restriction sites at the 3’ end were used to clone additional *pol* sequences downstream of XMRV *gag*. To map the activity of *pol* fragments unique NotI, KpnI, EcoRI, and XhoI sites were used to add and combine *pol* sequences downstream of XMRV *gag*. Initially XMRV *pol* was divided into three major fragments, encompassing nucleotides 2232–3457, 3457–5199, and 1611–2232*.* Fragment 2232–3457 was amplified with primers X04f and X04r and cloned KpnI-EcoRI downstream of XMRV *gag*. Fragment 3457–5199 was amplified with primers X20f and X20r and cloned EcoRI-XhoI downstream of XMRV *gag*. 1611–2232 was amplified with primers X07f and X07r and cloned KpnI-EcoRI downstream of XMRV *gag*. Fragments 2232–3457 and 3457–5199 were further truncated and cloned into XMRV *gag* with KpnI-EcoRI and EcoRI-XhoI, respectively. Fragment *2558*–*3456* was amplified with primers X08f and X04r, *2558*–*3307* was amplified with primers X08f and X09r, *2558*–*3155* was amplified with primers X08f and X10r, *2558*–*3007* was amplified with primers X08f and X11r, *2558*–*2858* was amplified with primers X08f and X12r, and *2558*–*2709* with primers X08f and X13r. *4715*–*5199* was amplified with primers X26f and X20r, and *4876*–*5199* was amplified with primers X27f and X20r.

Another construct included pol sequences from 4543 to 5199 and was used to mutate splice acceptor and donor sites. Potential splice donor sites were predicted using NetGene2 (http://www.cbs.dtu.dk/services/NetGene2/). The single point mutations in SA and SD were generated by overlapping PCR. We amplified two PCR products to mutate the SA site using primers X20f and X21r, and X21f and X20r. Primers X20f and X20r were used to fuse these two PCR products.

Moloney murine leukemia virus (MLV, accession number AF033811) *gag-pol* was generated by PCR in 3 fragments. The first fragment was cloned NheI-XhoI and the second fragment XhoI-EcorV, both into pcDNA3.1(−). The third fragment was cloned EcorV-NotI into pcDNA3.1(+). The first and the second fragment were subcloned as a NheI-EcoRV fragment into pcDNA3.1(+) that already contains the third fragment.

MLV *gag-pol*^*FS*^ has a frameshift at the 5’ end of MLV *pol*. This frameshift was induced by cutting the 5’ end of *pol* sequence at the unique restriction site XcmI and made blunt-ended with the Klenow fragment of DNA polymerase. pcMLV*gag* encompasses MLV *gag* as an NheI-NotI fragment and MLV *gag* 2232*–*3457 with MLV *pol* fragment 2232–3457 cloned XhoI-XbaI downstream of MLV *gag*.

An NXF1-binding domain (γ-CTE) was identified within *pol*. The secondary structure of a 100 nucleotide *pol* fragment including a putative NXF1 binding domain was modeled using the mfold program (http://mfold.rna.albany.edu/?q=mfold/RNA-Folding-Form). The γ-CTE, multimerized four times or eight times, was synthesized by GenScript, and subcloned downstream of gag. 4x X-CTE TBD4 and 8x X-CTE TBD4 contains mutations of 4 nucleotides within the critical NXF1-binding domain. The plasmid HIVgp1xCTE was provided by Dr. Michael Malim.

RNA from 293 T cells was reverse transcribed from RNA to complementary DNA (cDNA) by standard methods. The specific primer T01r was applied to obtain NXF1 cDNA and the T03r to obtain NXT2 cDNA. We amplified the cDNA with primers T01f and T01r to clone NXF1 XbaI-NotI into pcDNA3.1(−) Strep/Flag. The primers T02f and T02r were used to amplify NXT2 and clone it NheI-NotI into pcDNA3.1(−) Strep/Flag. The primers T03f and T03r were used to amplify SRp20, and the primers T06f and T06r to amplify SRp40. The primers T04f, T05f and T05r were used to amplify the two mutants SRp20ΔRRM and SRp20R3A, respectively.

For the knockdown vector cloning we used an shRNA design program (http://cancan.cshl.edu/RNAi_central/RNAi.cgi?type=shRNA). Three miRNA-based shRNA targeting sequences were designed against the NXF1/TAP transcript. Three 97-mer oligonucleotides were synthesized and PAGE purified, NXF1-1, NXF1-2, and NXF1-3 (sequences in Table 1), The 97-mer oligonucleotides were then amplified by PCR using primers: miR-30f and miR-30r. The PCR reaction was carried out with AccuPrime Pfx SuperMix, 1 mol/L Betaine (Sigma-Aldrich), 0.4 μmol/L each primer, and 100 ng 97-mer oligonucleotide template. The PCR product was column purified, digested with XhoI and EcoRI, and ligated in pAPM to create the pAPM-NXF1 knockdown constructs (NXF1-1, NXF1-2 and NXF1-3). The function of each NXF1 KD vector was checked by generating stable, puromycin-selected HEK293T cells. Efficiency of the NXF1 knockdowns was assessed by western blot for NXF1. NXF1-3 was selected for further experiments since it gave the most potent knockdown.

### Generation of NXF1 KD cells and rescue of the NXF1 protein

To generate stable microRNA-based shRNA KDs, HEK293T cells were transduced with pAPM microRNA-based shRNA vectors targeting either control or NXF1 mRNA (NXF1-1, NXF1-2, and NXF1-3). We transfected HEK293T cells with a DNA-mix containing 2.0 μg of pAPM (that contains the 97mer oligo), 1.4 μg of psPAX2 (packaging vector), and 0.7μg of pMD2G (envelope) on day 1. Then we collected the supernatent and filtered it through a 0.45 μm syringe filter. We removed 800 μl medium per well of the prepared HEK293T cells (6-well plate) and then added 800 μl of the supernatant (containing the virus) per well. 12 hrs after transduction, the cells were transfected with the plasmids of interest, using the Calcium Phosphate. 7 hrs after transfection, the cells were selected with 8 μg/mL puromycin. To generate the NXF1 rescue cells, HEK293T NXF1 KD and control KD cells were co-transfected with the plasmid of interest and the pcNXF1 expression vector, respectively. 7 hours after transfection, the cells were selected with 8 μg/mL puromycin for 36 hours and assayed for KD and protein rescue by SDS-PAGE⁄ western blot.

### Standard PCR

Standard PCR reactions were performed using either the High fidelity PCR system (Roche) or the AccuPrim Pfx system (Invitrogen), using specific primers at 0.2 μM and 0.4 μM, respectively. The template for the PCR reaction was either a plasmid or cDNA. The PCR reaction was set up according to the manufacturer’s instructions in Eppendorf Mastercycler.

### RT-PCR

The primers RT01f and RT01r (Table 2) were designed at the 5’ end of XMRV *gag* with an amplicon of 83 nt to detect XMRV constructs. The primers Rt02f and RT02r (Table 2) within MLV *gag* encompass a stretch of 88 nt and detects MLV constructs. The RNA expression levels were normalized against β-actin, using the primers RT03f and RT03r (Table 2). Initially cells were trypsinized, harvested and washed twice with PBS. 3.5 × 10^6^ cells of each sample were applied for Western Blot analysis to check total and cytoplasmic fractionation. The immunoblotting was conducted using anti-HSP90 and anti-Histone H3 antibodies as cytoplasmic and nuclear markers, respectively. Concomitant 3.5 × 10^6^ cells of each sample were resuspended in 175 μl of lysis buffer, and incubated on ice for 5 min. The sample was pelleted by centrifugation at 500 × g at 4°C for 5 min. Afterwards the supernatant was transferred to a new tube. Subsequent steps, for total and cytoplasmic fractionation, were performed using the RNeasy MINI Kit (Qiagen) following the manufacturer’s instructions.

**Table 2:** **Oligonucleotides used for qRT-PCR**

**Primer name**	**Primer sequence**
RT01f	5’-GTAACTACCCCTCTGAGTCTAACCT-3’
RT01r	5’-CTTCTTGACATCCACAGACTGGTT-3’
RT02f	5’-GTGGAGAAGCGACCCCTGCG-3’
RT02r	5’-GAATGCCTGCGAGGTAGTGGAG-3’
RT05f	5’- TGAGCTGCGTGTGGCTCC-3’
RT05r	5’- GGCATGGGGGAGGGCATACC-3’

After isolating total and cytoplasmic RNA the RT-PCR reaction was set up using 100 ng of template per reaction, 0.1 μM of each primer pair, and 2 x reaction buffer. Thermal cycler conditions used were 20 min RT reaction at 42°C, 5 min hot-start Taq activation at 95°C and 35 cycles of amplification. Each amplification cycle was composed of 5 s denaturation at 95°C, 5 s annealing at 58°C, 15 s extension at 72°C, 7 s acquisition at 83°C, using the lightcycler; 5 sec denaturation at 95°C, 5 sec annealing at 55°C, 20 sec extension at 72°C, 11 sec acquisition at 83°C. RT-PCR reactions were performed using the CFX96 thermal cycler (Biorad).

### Western blot

HEK293T cells were trypsinized and harvested by centrifugation for 5 min at 3000 rpm (Biorad 5418, FA-45-18-11, Standard rotor). The supernatant was removed and the cell pellets were lysed in an appropriate volume of RIPA buffer (1x PBS, 1% Nonidet P-40, 0.5% Na-deoxycholate, 0.05% SDS) for 30 min on a rotator. Subsequently, the lysed cells were centrifuged at maximum speed for 20 min. The supernatant was transferred into a new tube, mixed with 2x Laemmli buffer, heated for 5 min at 95°C and then resolved by SDS-PAGE. To analyze virus like particles we harvested the supernatent of a 10 cm dish of transfected 293 T cells, fltered the culture supernatant through a 0.45 μm filter. 9 ml of supernatent were put on 12 ml of 25% sucrose cushion and ultracentrifuged with a Beckman SW28 rotor at 26,000 × *g* for 1.5 hrs. The pellet was resuspended in 55 μl of phosphate-buffered saline (PBS) and analyzed by immunoblotting with an anti-CA antibody (Table 3).

**Table 3:** **Antibodies used in this study**

**Antibodies**	**Source**
Rat anti-p30 hybridoma	NIH AIDS Research and Reference Reagent Program, contributed by Bruce Chesebro
Rat Anti-SSFV Env (7C10)	Provided by Dr. Sandra Ruscetti, NCI
Anti-FLAG M2	Sigma Aldrich
Mouse Anti-TAP (NXF1)	BD Transduction Laboratories
Anti-rat HRP conjugated	Santa Cruz Biotechnology
Anti-mouse HRP conjugated	Santa Cruz Biotechnology

### Northern blot

HEK293T cells were trypsinized and harvested 48 h after transfection. Subsequently cells were washed twice with PBS. 3.5 × 10^6^ cells were resuspended in 175 μl of lysis buffer (50 mM Tris–HCl pH 8.0, 140 mM NaCl_2_, 1.5 mM MgCl_2_, 0.5% NP40, 1 mM DTT, 1000 U/ml RiboLock Fermentas) and incubated on ice for 5 min. The lysed cells were pelleted by centrifugation at 500 × *g* at 4°C for 5 min. Afterwards the supernatant was transferred to a new tube. We added 600 μl RLT Buffer (RNeasy MINI Kit, Qiagen). Then we added 430 μl to the homogenized lysate. 700 μl of the sample were transferred to an RNeasy spin column, and centrifuged for 15 s at ≥8000 × g. This step was repeated with the remaining sample. We added 500 μl RW1 to the RNeasy spin column and centrifuged again for 15 s at ≥8000 × g. Then we added 500 μl Buffer RPE twice to wash the spin column membrane for 15 s at ≥8000 × g and 2 min at ≥8000 × g, respectively. Then we placed the RNeasy spin column in a new 2 ml collection tube and centrifuged at full speed for 1 min. Finally we placed the RNeasy spin column in a new 1.5 ml collection tube and added 30 μl RNase-free water directly to the spin column membrane, and centrifuged for 1 min at ≥8000 × g to elute the RNA.

An RNA probe was generated by cloning XMRV *gag* into pBS KS(+). The plasmid was linearized using KpnI and incubated with T7 RNA polymerase and (α-^32^P-UTP) for two hours at 37°C using a protocol provided with the T7 RNA Polymerase (Fermentas).

Total and cytoplasmic RNA were separated on an agarose-formaldehyde gel (1x MOPS, 3,7% formaldehyde, 1% agarose) for 4 hrs at 90 V, 60 mA. As a marker the RiboRuler™ High Range RNA Ladder, 200–6000 bases (Fermentas), was used. We added 2x RNA loading dye containing ethidium bromide to stain the RNA. After electrophoresis the gel was incubated in 0.05 N NaOH buffer for 10 min. The gel and Hybond N Nylon membrane (Amersham) were then equilibrated in 0.5× TBE buffer for one hr. The transfer was performed in a Trans-Blot Semi-Dry Electrophoretic Transfer Cell (Biorad) for 35 min (3 mA/cm^2^). RNA crosslinking was conducted with a Stratalinker. Subsequently the membrane was hydrated in RNase-free water before adding the prehybridization buffer for two hrs at 68°C. Hybridization was performed by adding the (α-^32^P-UTP) labeled RNA probe for 16 hrs at 68°C. Afterwards the membrane was washed three times. The first wash (0.5 x SSC, 1% SDS) was at room temperature whilst the second and third washes (1× SSC, 1% SDS) were at 68°C. Blots were exposed to film and subsequently quantified on a Phosphorimager Typhoon FLA 7000 using the Image Quant TL analysis software.

### Polyribosome profile

48 h post-transfection of 2 × 10^7^ 293 T cells, cycloheximide was added to the tissue culture medium (50 μg/mL final concentration) for 5 min. The cells were then harvested by trypsinization and washed twice with cold PBS. Both the trypsin solution and the PBS also contained 50 μg/mL cycloheximide. The cell pellet was then resuspended in 400 μl of cold lysis buffer (50 mM Tris–HCl at pH 7.4, 100 mM KCl, 1.5 mM MgCl_2_, 1 mM DTT, 1 mg/ml Heparin, 1.5% NP40, 100 μg/ml cycloheximide, protease inhibitor cocktail Roche 100 μl/ml, 100 U RiboLock Fermentas), and incubated for 15 min on ice before centrifuging for 10 min at 12,000 × *g* at 4°C to remove the nuclei. The supernatant was loaded onto gradients of 15% to 55% sucrose (w/v). After ultracentrifugation in a Beckman SW41 rotor for 3 hrs at 210,000 × *g* at 4°C, the gradient was analyzed using an ISCO UA-6 collector. 2 μl of 20 μg/μl glycogen was added to each fraction which were then extracted with TriReagent as follows: after incubation on ice for 15 min, 300 μl of chloroform was added followed by centrifugation for 2 min at 12,000 × g. The upper phase was collected into a new tube and 700 μl of isopropanol was added. The mixture was stored for 30 min at −20°C. The RNA was pelleted at 12,000 × g for 10 min at 4°C. The supernatant was removed and the RNA was washed twice with 1 ml of ethanol. After removing the ethanol and air drying the pellet, the RNA pellet was resuspended in 20 μl of RNase-free H_2_O. After DNAse I (NEB) treatment, 100 ng was then used as template in the reverse transcriptase–polymerase chain reaction (RT- PCR).
